# Utilization of Dairy By-Products as a Source of Functional and Health Compounds—The Role of Ovine Colostrum and Milk Whey on Chronic Myeloid Leukemia Cells

**DOI:** 10.3390/foods12091752

**Published:** 2023-04-23

**Authors:** Carlotta Ceniti, Rosa Luisa Ambrosio, Jessica Bria, Anna Di Vito, Bruno Tilocca, Aniello Anastasio, Domenico Britti, Valeria Maria Morittu, Emanuela Chiarella

**Affiliations:** 1 Department of Health Sciences, University “Magna Græcia” of Catanzaro, C, 88100 Catanzaro, Italy; tilocca@unicz.it (B.T.); britti@unicz.it (D.B.); morittu@unicz.it (V.M.M.); 2 Interdepartmental Center Veterinary Service for Human and Animal Health, CISVetSUA, University “Magna Græcia”, 88100 Catanzaro, Italy; 3 Department of Veterinary Medicine and Animal Production, University of Naples Federico II, 80137 Naples, Italy; rosaluisa.ambrosio@unina.it (R.L.A.);; 4 Laboratory of Morphology and Tissue Cell Biology, Department of Experimental and Clinical Medicine, University “Magna Græcia”, 88100 Catanzaro, Italy; jessica.bria@studenti.unicz.it (J.B.); divito@unicz.it (A.D.V.); 5 Laboratory of Molecular Haematopoiesis and Stem Cell Biology, Department of Experimental and Clinical Medicine, University “Magna Græcia”, 88100 Catanzaro, Italy

**Keywords:** food safety, valorization, food by-product, colostrum, whey, milk, K562 cells, nutraceutical

## Abstract

Nowadays, the search for food products that promote consumers’ health has gained interest, and dairy by-products, due to their biological quality, could have a prominent position among products with health benefits. However, little is known about their activity on cancer cells. This study aimed to provide evidence about the effect of ovine colostrum and milk whey on K562 cells, a model of the human chronic myeloid leukemia cell line. The exposure of K562 cells to a single administration of sheep by-products at different concentrations for three days and three treatments for three days was carried out. Using a flow cytometric approach, we found that CD235a expression remained stable in the cells exposed to ovine whey (milk and colostrum) at concentrations ranging from 1 ng/mL to 100 μg/mL, after three days from one or three administrations, respectively. A significant reduction in fluorescent cells was observed in the populations exposed to 1 mg/mL of both milk and colostrum at the same time points. In these conditions, the size and granularity of the leukemic cells also changed, with a substantial reduction in the number of actively dividing cells in the S phase of the cell cycle. This phenomenon was highlighted by the Annexin V/PI cytofluorimetric test, which is able to provide quantitative results regarding the population of cells in early or late apoptosis or necrotic cells after exposure to a single dose or three doses of colostrum or sheep whey for three days, respectively. This report showed that both colostrum and milk whey were able to modify the phenotypic profile and cell cycle of the K562 cell line, inducing apoptosis at the highest concentration.

## 1. Introduction

Food-grade animal by-products are produced during industrial processing, and they are usually utilized for animal feed purposes or other low-value products [1]. Moreover, expired milk, cheese whey, colostrum, and other dairy industry by-products obtained from the dairy processing of various products could contribute to environmental pollution [2]. The revalorization of these by-products may lead to the development of a high-benefit product in a contest with human health, also including applications in non-food industries [3]. In this work, we focused on the biological potentials and possible valorization of colostrum and milk.

Colostrum is the first mammary secretion after parturition, and in subsequent days, it changes into standard milk [4]. In ruminants, this biological fluid ensures the growth, development, and immune support of newborns in early life [5]. It is well known that this “first milk” is significantly richer in valuable sources of immunoglobulins, biologically active peptides, components, and growth factors than milk [6,7]. To date, colostrum, as defined by Regulation (EC) No 853/2004, is not considered human food [8], and it is usually stored and employed as animal feed or treated as waste. Functional foods, which show a beneficial impact on human health and come from natural sources or by-products, especially of animal origin, are becoming increasingly important; in the last few decades, researchers have underlined the great importance of colostrum in this trade [9,10]. Ewe colostrum is rich in nutrients, bioactive compounds, immunoglobulins, peptides, and lipids and may have an important role as a constituent in functional dairy products [11,12,13]. Besides colostrum, the potential application of milk or cheese whey for new food products that promote consumers’ health has raised interest. Whey, a greenish-yellow liquid, is a by-product of cheese or casein manufacturing and varies in content according to the milk and cheese production process [2,14]. Recently, Pires and colleagues [15] revised the literature regarding cheese whey and second cheese whey, the by-product resulting from cheese production, in order to collect information on treatment processes and potential applications in human health. The author underlined that research must be carried out in order to give the dairy food sector valuable solutions aimed at enhancing their by-products, providing further economic valorization by their incorporation in food formulation, and increasing their efficiency and economic gain by reducing costs with their disposal and, thus, preventing environmental pollution. Thanks to its richness in nutritional components and the functional properties of its proteins, sheep milk whey was a target of investigation in order to find alternative methods of properly delivering bioactive peptides obtained from its hydrolysis in food systems through the use of liposomes [16]. More recently, experimental research has underlined the pharmacological potential of dairy whey. Cereda and colleagues (2019) evaluated the supplementation of whey protein in malnourished cancer patients to improve their energy balance and muscle mass [17]. In a very interesting work, Cacciola and colleagues [18] investigated the effect of a delactosed buffalo milk whey on tumor (colon cancer mouse xenograft tissues) progression in vivo, focusing on down- and up-regulation of the molecular players of anticancer mechanisms, demonstrating the potential role of whey as a source of biomolecules of food origin in novel strategies for the treatment of colon cancer. Thus, identifying dietary ingredients, natural food, or their bioactive compounds, especially from by-products, that have anticancer activities may lead to new therapeutic strategies. In this scenario, milk and colostrum could represent a promising biological source and are excellent candidates that should be investigated with further studies.

Here, we first evaluated the impact of two ovine by-products, colostrum, and milk whey, respectively, on K562 cells, a human model of chronic myeloid leukemia (CML). Chronic myeloid leukemia is a rare hematologic malignancy characterized by the expansion of the transformed hematopoietic stem or myeloid progenitor cells. Usually, this blood disorder progresses slowly and is genetically sustained by the reciprocal translocation, t(9;22)(q34;q11), giving rise to the Philadelphia chromosome (Ph 1), a molecular hallmark of CML [19,20].

For this purpose, we used a flow cytometry approach to analyze the CD235a expression, the typical marker of K562 cells, as well as the potential effects of ovine colostrum or milk whey on the cell cycle. Finally, the cytotoxicity induced by these sheep products was analyzed by investigating phosphatidylserine exposure and necrosis.

## 2. Materials and Methods

### 2.1. Milk and Colostrum Collection and Whey Preparation

Milk and colostrum were collected from an ovine dairy farm located in the area of Catanzaro, which contained 60–70 lactating animals (Sarda ewes) in a variety of lactation stages. The ovine milk was collected from a bulk tank after 3 days. For pool preparation, the colostrum of seven animals was collected within 8 h of parturition. Both colostrum and milk were stored at 4 °C and shipped to the laboratory of the University Magna Graecia of Catanzaro. The cream was separated by centrifugation at 3500× *g* (4 °C, for 30 min), and the fat was removed. Then, whey from each sample was obtained, as described by [21,22]. Briefly, a 10% rennet solution (100% chymosin; 200 international milk-clotting units/mL; Hansen Standard Chy-Max Plus 200, Chr. Hansen, Hørshoghm, Denmark) was added until curd formation was achieved; then, orthogonal vertical cuts, using a stainless-steel spatula, were made in order to collect the whey. Before the subsequent analysis, the whey (both colostrum and milk) was collected and filtered using a 0.45-µm syringe filter (Minisart, Sartorius Stedim Biotech GmbH, Göttingen, Germany). The proteins (mg/mL) were quantified for both ovine colostrum and milk whey with a spectrophotometer using a Quick Start^TM^ Bradford for protein assays (Bio-Rad) by the reading of the samples in triplicate at 595 nm. The amount of protein was calculated by interpolation of the experimental values with standard proteins to a known concentration.

### 2.2. Cell Culture and Cell Treatment with Ovine Colostrum or Milk Whey

The human hematopoietic cell lines, K562, were obtained from ATCC (CCL-243) and were cultured as described in [23,24]. Briefly, K562 cells were grown in RPMI (Invitrogen Corporation; Cat No. 23400-021), supplemented with 10% FBS, 50U of penicillin, and 50 μg/mL of streptomycin, and maintained in a 5% CO_2_ incubator at 37 °C. For all experiments, K562 cells (passage number 25–30) were seeded at a density of 2 × 10^5^ cells per well in a 12-well plate and then stimulated with ovine colostrum (1 ng/mL, 10 ng/mL, 100 ng/mL, 1μg/mL, 10 μg/mL, 100 μg/mL, and 1 mg/mL) or ovine milk whey (1 ng/mL, 10 ng/mL, 100 ng/mL, 1 μg/mL, 10 μg/mL, 100 μg/mL, and 1 mg/mL) for three days. In particular, one set of cells was treated with a single administration of colostrum or milk whey at various concentrations and analyzed after 72 h, while another was subjected to three daily administrations and always analyzed after 72 h.

### 2.3. Effect of Milk Whey and Colostrum Whey on K562 Cell Surface Antigen

The expression of glycophorin A (CD235a) on the K562 cell surface was determined by flow cytometry, as described in [25]. The cells were washed with PBS and stained with a PE fluorescent-conjugated antibody for CD235a (Miltenyi Biotec, Bergisch Gladbach, Germany) in the dark for 30 min on ice. Afterward, the samples were washed, re-suspended in 300 μL of cold PBS, and acquired on a BD FACscan™ II. The percentage of CD235a-positive cells was determined by FlowJo software 8.8.6 (Becton Dickinson, Milan, Italy).

### 2.4. Analysis of the Cell Cycle

The cell cycle was performed by staining cells with propidium iodide (PI), a vital nuclear dye that intercalates with DNA, as described in [26]. Briefly, 2 × 10^5^ cells were collected in a 5 mL polystyrene tube and washed twice with cold PBS. Cells were fixed by vortexing and slowly adding, drop-by-drop, 1mL of ice-cold 70% ethanol to the pellet. Cells were incubated for 40 min in the dark at 4 °C and washed three times with cold PBS. Subsequently, the cell pellet was treated with RNase (40 μg/mL) (Sigma-Aldrich, Milan, Italy) for 30 min at room temperature and finally stained with PI (50 μg/mL) for 10 min at room temperature. The propidium iodide (PI) signal was read by a BD FACscan™ II, using 488-nm excitation; the doublets were gated out using the area vs. width. The data were analyzed by FlowJo software 8.8.6 with histograms representing the proportion of cells in each stage of the cell cycle based on variations in DNA content.

### 2.5. Annexin V/Propidium Iodide (PI) Staining Assay

The detection of live, necrotic, and apoptotic cells was performed by using an FITC Annexin V Apoptosis Detection kit with PI (Biolegend #640914), as described in [27]. Briefly, 2 × 10^5^ cells were subjected to single or repeated daily treatment with ovine colostrum or ovine whey at concentrations of 100 μg/mL or 1 mg/mL and were then analyzed after three days. Cells were centrifuged and washed with PBS 1×. An amount of 100 µL of cell suspension was stained with 5 µL of FITC Annexin V and 10 µL of propidium iodide solution according to the manufacturer’s instructions. The samples were then gently vortexed and incubated for 15 min at room temperature (25 °C) in the dark. An amount of 400 µL of an Annexin V Binding Buffer was added to each tube for flow cytometry acquisition (BD FACScan II™). The different fractions of cells in early apoptosis (An+/PI−) and late apoptosis/necrosis. (An+/PI+, An−/PI+) were evaluated by a proper gating strategy using FlowJo software 8.8.6.

### 2.6. Statistical Analysis

For statistical analyses, *t*-tests were applied by Microsoft^®^ Excel^®^ 2010 (Redmond, WA, USA); *p* < 0.05 was taken as a level of significance.

## 3. Results

### 3.1. Effect of Ovine Colostrum on K562 Cell Surface Profiling

**Figure 1:**
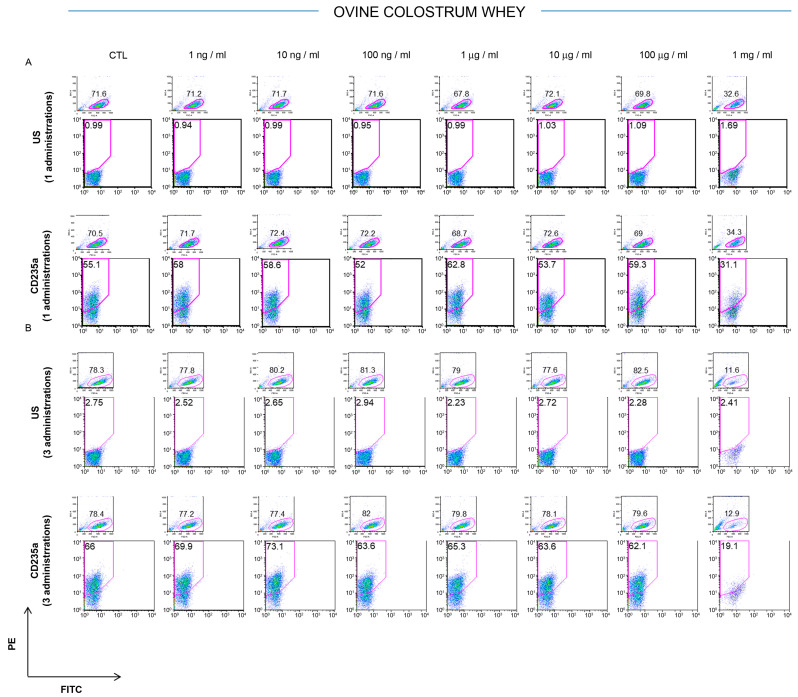
Analysis of CD235a cell surface antigen in K562 exposed to ovine colostrum whey. The K562 phenotypic pattern for CD235 expression was investigated by FACS analysis after three days from one (**A**) or three administrations of ovine colostrum whey (**B**) at different concentrations (1 ng/mL, 10 ng/mL, 100 ng/mL, 1 μg/mL, 10 μg/mL, 100 μg/mL, and 1 mg/mL). Data are collected from three different experiments.

**Figure 2:**
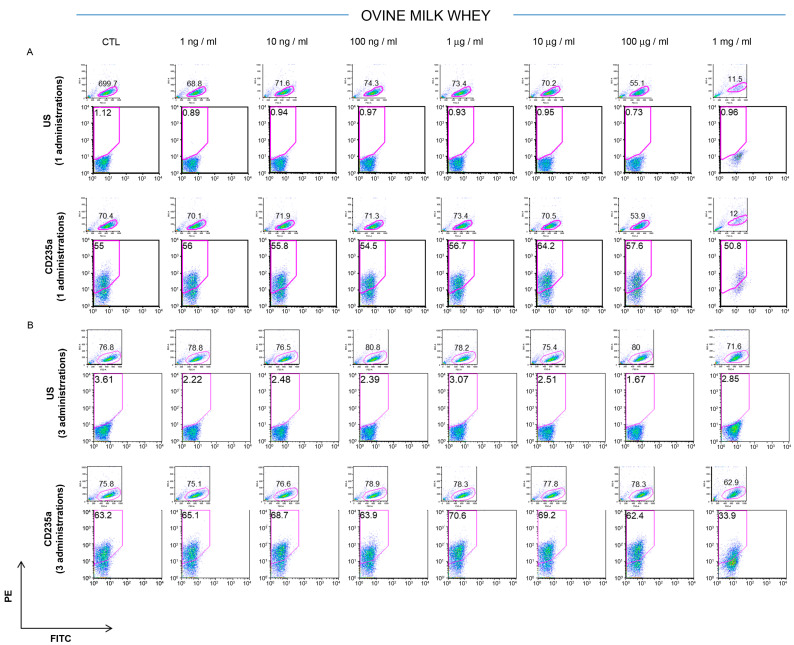
Cytofluorimetric analysis of K562 exposed to ovine bulk milk whey. The K562 positivity for the CD235a cell surface antigen was assessed three days after one (**A**) or three administrations (**B**) of ovine bulk milk. Cells were treated by increasing concentrations of ovine bulk milk (1 ng/mL, 10 ng/mL, 100 ng/mL, 1 μg/mL, 10 μg/mL, 100 μg/mL, and 1 mg/mL) and then analyzed by FACS. Three independent experiments were performed.

The potential effects of ovine colostrum were assessed on the K562 cells’ phenotypic signature after 3 days from a single or once-daily repeated administration and compared with those of the ovine serum. The untreated cells were used as a control. The CD235a antigen, also known as glycophorin A, is a type I single-pass membrane protein typically associated with erythrocytes and erythroid precursor cells. It is a sialoglycoprotein with a relevant role in preventing cell fusion and, so, the improper aggregation between red blood cells during circulation [28]. The CD235a surface antigen detection on the K562 cells remained largely stable (about 60%) after the treatment with 1 ng/mL, 10 ng/mL, 100 ng/mL, 1 μg/mL, 10 μg/mL, and 100 μg/mL of ovine colostrum, respectively, but a significant reduction (CTL 55.1% vs. 1mg/mL treated 31.1% of CD235a-positive cells) was observed when the cells were cultured in the presence of 1 mg/mL of colostrum (Figure 1A). It is remarkable that this reduction was already observable after one single administration, and it became more evident when the cells were exposed to three doses of 1mg/mL of colostrum for three consecutive days (CTL 66% vs. 1mg/mL for three days 19.1% of CD235a-positive cells). In this case, the percentage of CD235a was reduced by 71.07% (Figure 1B). These results were due to reduced cell viability that emerged from the ratio of the forward versus the side scatter plot in each ancestry, showing the segregation of damaged cells. A more attenuated trend was recorded when the K562 cells were cultured in the presence of different concentrations of ovine milk whey (1 ng/mL, 10 ng/mL, 100 ng/mL, 1 μg/mL, 10 μg/mL, 100 μg/mL, and 1 mg/mL). In this case, the expression of CD235a was reduced in the cells treated with 1 mg/mL of ovine whey compared to the lowest concentrations after one or three doses, respectively (Figure 2A,B). In particular, a significant reduction in the CD235a expression of 46.3% (CTL, 63.2%, vs. 1mg/mL for three days, 33.9%) is appreciated after three daily treatments with 1 mg/mL of milk whey compared to the cells subjected to only one treatment (CTL 55% vs. 1mg/mL treated once 50.8% of CD235a-positive cells).

### 3.2. Effect of Ovine Colostrum and Ovine Milk Whey on Leukemia K562 Cell Proliferation

**Figure 3:**
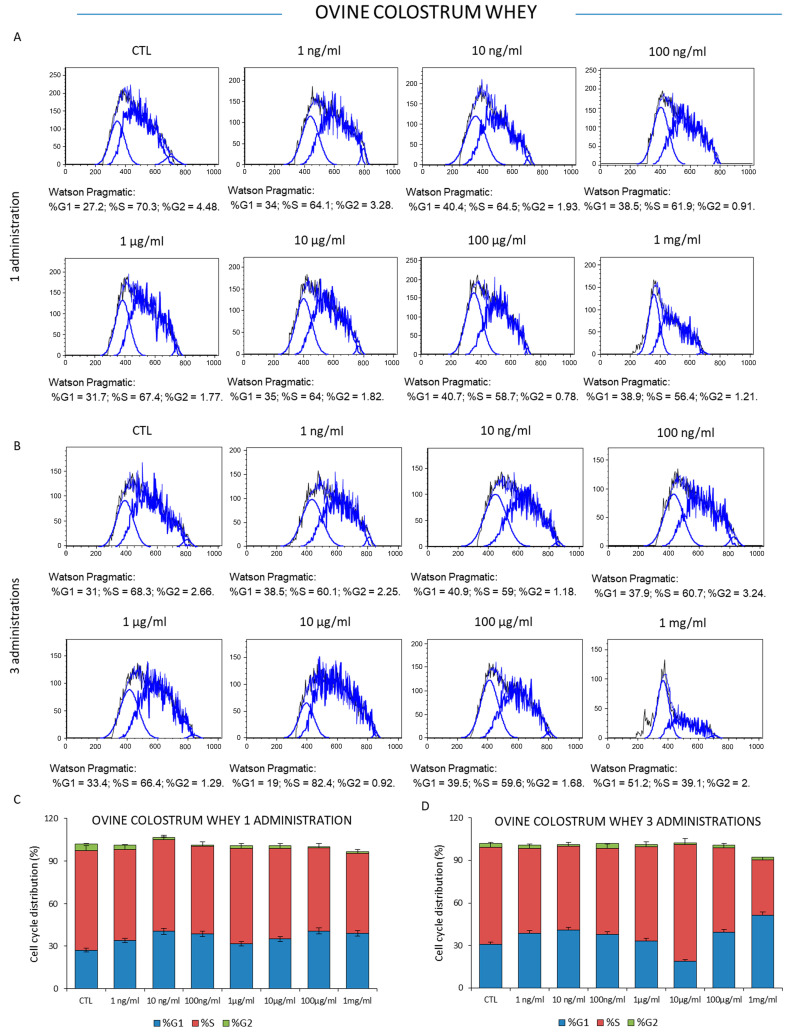
Analysis of K562 cell cycle distribution after colostrum whey administration. The number of cells across the G0/G1, S, and G2/M phases of the cell cycle was determined following one (**A**) or three treatments (**B**) with colostrum whey for three days at concentrations of 1 ng/mL, 10 ng/mL, 100 ng/mL, 1 μg/mL, 10 μg/mL, 100 μg/mL, and 1 mg/mL, respectively. (**C**,**D**) Histograms show the percentage of K562 cells in the three stages of the cycle (G1 vs. S vs. G2/M) after three days of exposition to one or three doses of ovine colostrum whey at concentrations of 1 ng/mL, 10 ng/mL, 100 ng/mL, 1 μg/mL, 10 μg/mL, 100 μg/mL, and 1 mg/mL, respectively. The experiments were repeated in triplicate.

**Figure 4:**
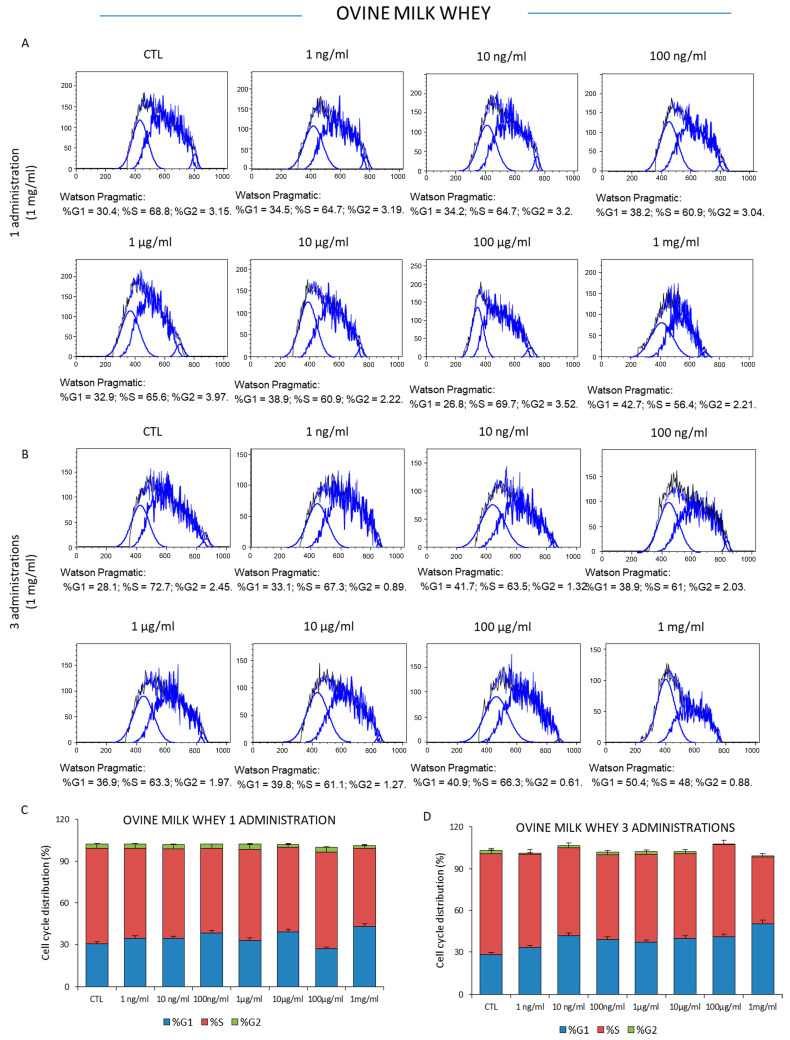
Analysis of K562 cell cycle distribution after bulk milk whey administration. The number of cells across the G0/G1, S, and G2/M phases of the cell cycle was determined following one (**A**) or three treatments (**B**) with bulk milk whey for three days at concentrations of 1 ng/mL, 10 ng/mL, 100 ng/mL, 1 μg/mL, 10 μg/mL, 100 μg/mL, and 1 mg/mL, respectively. (**C**,**D**) Bar graphs represent the percentage of cells in G1, S, and G2/M stages of the K562 cell cycle after three days of exposition to one or three doses of ovine milk whey at concentrations of 1 ng/mL, 10 ng/mL, 100 ng/mL, 1 μg/mL, 10 μg/mL, 100 μg/mL, and 1 mg/mL, respectively. The experiments were repeated in triplicate.

To investigate the effect on the proliferative properties of ovine colostrum, the K562 leukemia cells were treated one or three times with increasing concentrations of colostrum (1 ng/mL, 10 ng/mL, 100 ng/mL, 1 μg/mL, 10 μg/mL, 100 μg/mL, and 1 mg/mL) for 72 h, and then the cell viability was measured. The results showed that sheep colostrum did not significantly affect the viability of mammalian K562 cancer cells at relatively low doses. Specifically, S phase fluctuations of the cell cycle were observable when the cells were treated with colostrum concentrations between 1 ng/mL and 100 μg/mL in response to one- or three-time administrations, respectively. However, the number of actively dividing cells, as well as the percentage of cells in the S phase, significantly decreased from 70.3% (the control condition) to 56.4% when the cells were treated with a single administration of 1 mg/mL of ovine colostrum for three days (Figure 3A–C). Interestingly, these results were accompanied by a sub-G0/G1 population at the left of the G0/G1 peak, indicating the presence of apoptotic cells, above all those detectable after 72 h from three administrations. In this case, the percentage of actively dividing cells was reduced from 68.3% (the control condition) to 39.1% (Figure 3B–D). Similarly, a single administration of sheep milk whey did not significantly affect the proportion of cells in the three interphases of the cell cycle at concentrations between 1 ng/mL and 100 μg/mL. This effect was reproducible when the K562 cells were the recipients of three doses of sheep milk whey, while a significant reduction in the S phase of the cell cycle was observed after the treatment with the highest milk whey concentration of 1mg/mL (Figure 4C,D). In this experimental context, the percentage of proliferative cells changed from 66.8% in the control cells to 56.4% in the cells exposed to a single milk whey dose (1 mg/mL) and 48% in those treated with three administrations (1 mg/mL) of this dairy by-product.

### 3.3. Cytotoxicity of Ovine Colostrum and Ovine Serum

**Figure 5:**
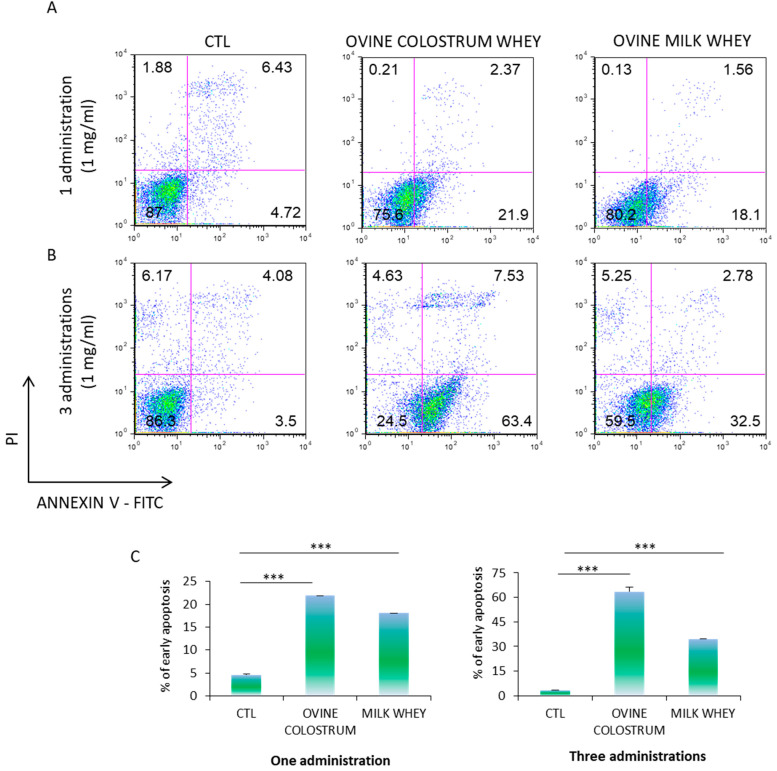
In proliferative conditions, K562 cells undergo apoptosis when treated with high concentrations of ovine colostrum and bulk milk. (**A**,**B**) CTL-K562 cells and K562 cells cultivated in the presence of one or three doses of ovine colostrum and bulk milk (1mg/mL) for three days were stained for both annexin V-FITC and PI to detect early apoptosis, late apoptosis, and necrosis by FACS. (**C**) The percentage of early apoptosis is represented as means ± SD from three different experiments. In each analysis, a *p*-value < 0.05 was considered statistically significant. ***, *p* < 0.001. CTL: control; PI: propidium iodide.

To investigate the cytotoxic effect of ovine colostrum on the K562 cells, the apoptosis level was measured by Annexin V-PE/PI staining. According to the CD235a expression and cell cycle analysis, we exposed the K562 cells to 1 mg/mL of ovine colostrum for three days, once again adopting the experimental scheme that provides for single or three daily administrations, respectively. The analysis revealed that the ovine colostrum treatment induced a significant increase in the frequency of Annexin V+ cells in both cell populations compared to those treated with ovine whey (Figure 5A–C). Moreover, the metachromatic shift from red to green fluorescence increased in a time-dependent manner in the ovine colostrum-treated cells, reflecting the biochemical changes specific for early apoptosis (21.9% in the samples exposed to a single ovine colostrum treatment for three days vs. 63.4% in the samples subjected to three daily ovine colostrum administrations for three days). These data resulted more attenuated at 72 h in the cells treated once or three times by ovine milk whey (18.1% in the cells exposed to one ovine colostrum treatment for three days vs. 32.5% in the samples exposed to three daily ovine colostrum administrations for three days). These data suggested that both ovine colostrum and ovine serum may play an important role in inducing apoptosis in K562 cells. The above results were confirmed by three independent analyses.

## 4. Discussion and Conclusions

Nowadays, consumers are increasingly interested in food quality and health promotion, and thus nutraceutical application for disease prevention is a topic of great importance for food industries; emerging strategies have aimed to identify biomolecules from food by-products and dairy by-products for health promotion are attracting attention. In this study, for the first time, we evaluated the effect of sheep milk whey and colostrum whey on K562 cells by testing their CD235a expression profile, cell cycle, and apoptosis. Our results showed that both ovine colostrum whey and milk whey impact K562 cell viability. In particular, the sheep colostrum whey exerted a greater apoptotic effect than that induced by sheep milk whey when leukemic cells were treated once or three times at a concentration of 1 mg/mL.

Regarding colostrum, several studies have investigated its potential for applications in human nutrition and health [6,29,30,31,32]. Recently, some authors focused on the anticancer activity of colostrum in humans [33], although the bibliography is still limited and focused on bovine colostrum. Do Carmo França-Botelho [34] explored the peptide colostrinin effect, suggesting proinflammatory activity that resulted in the stimulation of the activity of natural killer cells, inducing IL-6, IL-10, and TNF-alpha and inhibiting superoxide dismutase in the cancer patients. In 2016, Agarwal and Gupta [35] highlighted how the bioactive compounds of colostrum could help to prevent the growth of cancer cells and, among all the components, they focused their attention on lactoferrin, an iron-binding protein, which appears to have prominent anticancer activity. Purified lactoferrin from caprine colostrum was tested on various cancer cell lines, including those of lung, colon, cervix, stomach, and breast cancer cells via the colorimetric 3-(4,5-dimethylthiazol-2-yl)-2,5-diphenyltetrazolium bromide (MTT) assay, showing dose-dependent antiproliferative effects [36]. Sharma and colleagues (2019) [37] evaluated the in vitro anticancer potential of colostrum-derived lactoferrin from the Indian native zebu cow (Sahiwal); crossbred (Karan Fries) and commercially available lactoferrin from exotic cows showed that colostrum lactoferrin had the capacity to inhibit the growth of the cancerous cells MDA-MB-231 and MCF-7. Gibbons et al. (2015) [38] stated the increased cytotoxicity and decreased cell proliferation in MDA-MB-231 and MCF-7 human breast cancer cell lines upon iron-free and iron-saturated bovine lactoferrin treatment. Besides lactoferrin, other substances may have anticancer activity. More recently, Alsayed [39] has summarized anticancer activity in the treatment of several bovine colostrum components, including lactoferrin, conjugated linoleic acid (CLA), and alpha-lactalbumin. Although their anticancer activity has been confirmed, other studies are required to evaluate the appropriate dosages of the drugs, their therapeutic effects, and their long-term safety. Interestingly, in the present investigation, the antitumor effect of ovine colostrum is also manifested in chronic myeloid leukemia K562 cells. The reduction of cell viability was already observable after one single administration, and it became more evident when the cells were exposed to three doses of 1mg/mL ovine colostrum for three consecutive days, as showed expression of CD235a antigen. The expression of GlycophorinA is considered a valuable marker to study the this chronic myelogenus leukemic cell line, however further investigations will be needed to define K562 cells surface profiling after colostrum and milk whey treatment, including CD71 and CD44 markers [40,41].

In particular, from the ratio of forward versus side scatter plots in each ancestry, the segregation of damaged cells is evident. The results are confirmed by the Annexin V-PE/PI staining measurement that showed cells in early apoptosis. Specifically, after one administration, it was observed that about 21.9% of the cells were in early apoptosis on the third day. These results were increased when the cells were exposed to three administrations of ovine colostrum whey, resulting in 63.4% of apoptotic cells after three days.

Regarding milk whey, our results also underlined that ovine milk influenced K562 cell behavior. Cytofluorimetric analysis of K562 cells treated once or three times with 1 mg/mL of ovine bulk milk whey showed reduced viability, as emerged from the ancestry of the cells tested for CD235a expression, as well as from the cell cycle distribution in the cell cycle stages compared to the lowest concentration at the same times. In a very interesting study, Ji and colleagues analyzed the effect of the milk fat globule membrane, the membrane proteins, and lipids that surround the fat globules in milk, on human colon cancer HT-29 cells; the milk fat globule membrane from bovine, goat, buffalo, yak, and camel milk was investigated for the effect of the proliferation of human colon cancer HT-29 cells. The authors showed that the milk fat globule membrane induced cell death in a concentration-dependent and time-dependent manner; in particular, when the cells were cultured for 72 h in the presence of goat, buffalo, and bovine globule membranes at 100 μg/mL, the cell cycle was affected in the G0/G1 and S phase [42]. According to these data, here, we noticed a significant reduction in the S phase of the cell cycle after the treatment with the higher milk whey concentration (1 mg/mL). In the present investigation, the percentage of active dividing cells in the S phase of the cell cycle changed from 68.8% in the control cells to 56.4% in the cells exposed to a single milk whey dose (1 mg/mL) and 48% in those treated with three administrations (1 mg/mL).

Furthermore, cytotoxic, apoptotic, and cycle modulating properties of milk whey were also reported in colorectal cancer; D’Onofrio and colleagues tested the antiproliferative effect of whey from Mediterranean water buffalo (*Bubalus bubalis*) milk in colorectal cancer (CRC) cells, HT-29, HCT 116, LoVo, and SW480, demonstrating both its ability to affect the cell cycle via a reduction of the number of cells in the S phase, especially after 72 h, as well as the cytotoxic effect detected by the externalization of phosphatidylserine (PS) on the plasma membrane [43]. Our investigation fits well with these results in terms of the reduction of the cell cycle and induction of apoptosis. When K562 cells were exposed to 1 mg of ovine milk whey, we recorded about 18.1% of cells in early apoptosis. This effect was potentiated in the cells exposed to three administrations of ovine milk whey, resulting in 32.5% of apoptotic cells after three days.

In summary, we conclude that both ovine colostrum and whey negatively impact the viability of K562 cells when administered at 1 mg/mL of these dairy by-products for three days. In particular, we noticed the antileukemic effect of colostrum already following a single treatment for three days, and this turned out to be amplified after three administrations for three days. On the other hand, milk whey was found to be less efficient than colostrum. A possible cause for this difference in behavior may be due to the different compositions of colostrum and milk; thus, extensive investigation into this finding could prove very interesting.

Dairy by-products are expected to drive new markets in the functional food industry as food ingredients with beneficial properties, including anticancer activity. Moreover, the efficient utilization of dairy by-products can help reduce the costs of food waste generation by introducing a new waste product pathway that makes the dairy food industry more sustainable. Our results could have promising implications for the creation of new functional food ingredients. Several other studies should focus on resolving the limitations of our study; for example, it will be worthwhile investigating further which specific and molecular mechanism employed by a component of colostrum and milk whey plays the inhibitory role on K562 cells. Moreover, the technological transformation strategies aimed at preserving the observed effect, enabling its diffusion/utilization, and nutraceutical food ingredients should be further investigated.

## Data Availability

Data is contained within the article.
